# Pre- and postnatal safe sleep knowledge and planned as compared to actual infant sleep practices

**DOI:** 10.1186/s40621-023-00467-0

**Published:** 2023-10-26

**Authors:** Paula Valiño Ramos, Pamela J. Hoogerwerf, Penny K. Smith, Carolyn Finley, Uche E. Okoro, Charles A. Jennissen

**Affiliations:** 1 Department of Emergency Medicine, Roy J. and Lucille A. Carver College of Medicine, University of Iowa, Iowa City, IA USA; 2 Medical Scientist Training Program, Washington University in St. Louis, St. Louis, MO USA; 3 Injury Prevention and Community Outreach, University of Iowa Stead Family Children’s Hospital, University of Iowa, Iowa City, IA USA; 4 Iowa’s Statewide Perinatal Care Program, University of Iowa Stead Family Children’s Hospital, University of Iowa, Iowa City, IA USA; 5 University of Iowa Stead Family Children’s Hospital, University of Iowa, Iowa City, IA USA; 6 Department of Pediatrics, Roy J. and Lucille A. Carver College of Medicine, University of Iowa, Iowa City, IA USA

**Keywords:** Accidental suffocation, Cribs, Back, Infants, Mothers, Prenatal, Postnatal, Safe sleep, Sudden infant death syndrome, Sudden unexpected infant death

## Abstract

**Background:**

Our objectives were to compare safe sleep knowledge, attitudes and planned vs. actual infant sleep practices among expectant mothers before and after their infant’s birth and to determine whether differences (if present) were associated with any demographic variables.

**Methods:**

Study participants were surveyed at their 28-week prenatal and 6-week postpartum obstetric clinic visits from November 2019–February 2021. Due to COVID-19 pandemic cancellation of in-person postpartum visits, many participants received text messaging encouraging them to take the follow-up survey online. Frequency and comparative analyses were performed.

**Results:**

355 women (44%) completed both pre- and postnatal surveys. Many participants increased their safe sleep knowledge during the study. For example, of those who were unsure or thought it safe for a baby to sleep in a baby swing/bouncy seat, two-thirds (67/102, 66%) stated it was unsafe on the postnatal survey. In addition, many who were unsure or planned sleep practices considered unsafe prenatally reported utilizing safe sleep practices on their postnatal survey. For example, of those unsure or planning to use a crib bumper (17% of the total), almost all (88%) were not using one postnatally. Conversely, some participants who reported they would be following safe sleep practices prenatally were not doing so postpartum. For example, 13% of those stating they would place their child on their back reported using another sleep position on the postnatal survey. Certain demographics had higher proportions reporting this reversal for specific safe sleep practices. For example, non-Hispanic Whites (19%) as compared to other races/ethnicities (5%) and those with incomes ≥ $75,000 (21%) as compared with those with less income (9%) had higher proportions stating their infant would sleep in the same room but then reported postnatally they were sleeping in a different room, *p* = 0.0094 and *p* = 0.0138, respectively.

**Conclusions:**

We observed increases in safe sleep knowledge and that some participants followed safer sleep practices than they had planned. However, there were also participants who planned to use safe sleep practices prenatally who were not doing so after their baby’s birth. Our study identified demographics for which targeted safe sleep education and more effective interventions may be needed.

## Background

Sudden unexpected infant deaths (SUID) due to accidental suffocation and strangulation in bed, sudden infant death syndrome (SIDS) and unknown causes account for about 3,500 infant deaths in the USA each year (Moon et al. 2022). Nearly all these deaths occur during infant sleep or in the sleep environment. Despite increased knowledge of risks and protective factors (Moon et al. 2022), the overall rate of these sleep-related deaths has remained essentially unchanged for the past 20 years (Quinlan et al. 2018). SUID are the leading cause of post-neonatal mortality (> 28 days to 1 year of age) (Moon et al. 2022).

A high proportion of parents are uninformed about SUID and do not follow recommended infant safe sleep guidelines (Rohana et al. 2018; Ruiz Botia et al. 2020). However, studies have shown an increase in parental knowledge and compliance with recommended infant safe sleep guidelines after specific educational strategies (Rohana et al. 2018; Ruiz Botia et al. 2020; Hutton et al. 2017). The effectiveness of some public health interventions has also been evaluated (Olivera Olmedo et al. 1998). Still, there are very little data comparing the safe sleep knowledge, attitudes and planned/actual infant sleep practices of expectant parents prenatally with those postnatally.

The objective of our study was to determine whether mothers’ safe sleep knowledge, attitudes and infant sleep plans changed from before and after birth, and whether differences, if present, were associated with any demographic variables.

## Methods

### Survey development

The surveys were developed by members of the research team with input from the University of Iowa Stead Family Children’s Hospital Safe Sleep Task Force. For validation, the survey tools were administered in both a written and interview format to ten volunteers who were patients at a University of Iowa Hospitals and Clinics (UIHC) obstetrics clinic. Paper and in-person interview survey responses were compared for consistency. The reasons for any inconsistencies were identified and resolved. The University of Iowa Institutional Review Board approved this study.

### Study variables

Demographic variables included age, race/ethnicity, insurance (employer-based, privately purchased, Medicare/Medicaid, none), family income and number of children. Name and phone numbers of participants were also collected. For analysis, ages were grouped as 18–24, 25–29, 30–34 and ≥ 35 years. Race/ethnicity was self-reported. Given the low racial/ethnic diversity of the population, race/ethnicity was categorized as Non-Hispanic White, Hispanic/Latinx and Non-Hispanic Other. The latter two categories were combined for some analyses. Family income was grouped as $0–24,999, $25,000–49,999, $50,000–74,999, $75,000–99,999 and ≥ $100,000. Number of children categories included 0, 1 and ≥ 2 children.

Study participants were asked their level of confidence with providing a safe place for the baby to sleep as well as planned and actual infant sleep practices on the pre- and postnatal survey. Many sleep practices were queried but some of the primary ones included infant sleep location (same or different room where mother slept), sleep space (crib, bassinet, play yard versus other spaces considered unsafe) and sleep position (back, stomach or side).

Safe sleep knowledge was determined by asking whether it was safe or unsafe for a baby less than one year to sleep in a baby swing/bouncy seat, car seat, couch/sofa/recliner, bassinet, play yard, baby’s own crib, in bed with an adult or in bed with another child. Attitudes and knowledge about additional safe sleep practices were assessed by asking the level of agreement or disagreement with several statements using a 5-point Likert scale. On the postnatal survey, respondents were also asked from what sources they had heard, seen or read anything about infant safe sleep.

### Survey distribution

From November 2019 to September 2020, all pregnant women presenting for 28-week prenatal visits at UIHC-associated obstetrics clinics were surveyed. Trained clinic and nursing staff provided potential subjects the prenatal survey and research consent letter, if willing, to read and complete. Those under 18 years of age (legal age of consent in Iowa is 18 years) and those who could not read English were excluded from the study.

Starting January 2020, all women presenting to the UIHC obstetrics clinics for their 6-week postpartum visit were given a consent letter and the Safe Sleep postnatal survey regardless of whether they had completed a prenatal survey. The beginning date to collect postnatal surveys was selected to potentially include all women who had completed the prenatal survey. All surveys were placed in a designated file folder at the nurses’ station and locked in a cabinet at the end of the clinic day. Completed surveys were collected monthly by research team members.

With the onset of the COVID-19 pandemic, obstetricians began doing virtual postpartum visits rather than in-person. Therefore, those who completed the prenatal survey were contacted by text at about 6-week postpartum and provided a link to complete the postnatal survey online on Qualtrics™ after reading the consent letter. Two follow-up text reminders were also sent.

### Data analysis

Of the 1023 women eligible, 814 (80%) completed the prenatal Safe Sleep survey. Written survey responses were entered into Qualtrics™. LinkPlus software and manual methods were used to match participant’s pre- and postnatal surveys. Only participants with matching surveys (*N* = 355) were included in response analysis. Demographic comparisons were also done between participants who had completed both surveys and patients who had only completed the prenatal survey. Descriptive (frequencies) and comparative analyses, including bivariate (chi-square, Fisher’s exact test) analyses, were performed using SAS (previously Statistical Analysis System) 9.4 software and vassarstats.net. Fisher’s exact test was used for any comparison in which a cell had a predicted value of < 5. All p values were two-tailed, and a value < 0.05 was considered statistically significant. Missing data were not included in analyses.

## Results

A total of 355 participants completed both the prenatal and the postnatal survey (44% of those who completed the prenatal survey). Table 1 shows the demographic breakdown of study participants and a comparison with those who completed the prenatal survey only. For participants who completed both surveys, over four-fifths self-identified as non-Hispanic White and about one-half had incomes of $75,000 and greater. One-fifth were insured by Medicaid/Medicare, and 77% had employer-provided insurance. For 42% of participants, this pregnancy was going to be their first child.

**Table 1:** Demographics of study participants who completed both the 28-Week Prenatal Visit and the Postnatal Safe Sleep Survey (*N* = 355) and a comparison with the demographics of women who completed the 28-Week Prenatal Visit Safe Sleep Survey only (*N* = 459)

Variable	Completed both pre- and postnatal survey n (Col%)^a,b^	Completed prenatal survey only n (Col%)^a,b^	*p* Value
*Age*			0.0740
18–24 yrs	41 (12)	81 (19)	
25–29 yrs	114 (33)	134 (31)	
30–34 yrs	117 (34)	135 (31)	
≥ 35 yrs	76 (22)	85 (20)	
*Race/ethnicity*			< 0.0001
NH White	290 (82)	315 (69)	
Hispanic/Latinx	34 (10)	37 (8)	
NH Other	31 (9)	107 (23)	
*Family income*			< 0.0001
$0-$9,999	12 (4)	46 (11)	
$10,000–$24,999	28 (8)	64 (16)	
$25,000–$49,999	36 (11)	73 (18)	
$50,000–$74,999	51 (15)	68 (17)	
$75,000–$99,999	78 (23)	56 (14)	
≥ $100,000	132 (39)	105 (26)	
*Insurance*			< 0.0001
Medicare/Medicaid	69 (20)	191 (45)	
Through employer	273 (77)	216 (51)	
Privately purchased	2 (1)	15 (4)	
None	0 (0)	5 (1)	
*Number of children presently in family*			0.0309
0 Children	146 (42)	150 (35)	
1 Child	122 (35)	150 (35)	
≥ 2 Children	80 (23)	133. (31)	

*NH* non-Hispanic; *yrs* years

^a^Column total may not equal group N due to missing data

^b^Total column percentage may not equal 100% due to rounding

There were significant demographic differences between those who completed both the prenatal and postnatal surveys and those who completed the prenatal survey only. See Table 1. Women who completed both surveys had higher proportions of non-Hispanic Whites, of family incomes ≥ $75,000, of employer-provided insurance and of pregnancies being their first child.

On the postnatal survey, almost all participants stated that they felt “very confident” (80%) or “confident” (19%) in their ability to provide a safe place for their baby to sleep. See Table 2. This was significantly higher than that reported on the prenatal survey, *p* < 0.0001.

**Table 2:** Confidence in providing safe infant sleep and planned vs. actual infant sleep practices as reported by women that completed both the 28-Week Prenatal Visit and the Postnatal Safe Sleep Surveys (N = 355).^a,b^

*Reported confidence level*		Prenatal	Postnatal
Very confident		220 (64)	281 (80)
Confident		87 (25)	67 (19)
Somewhat confident		31 (9)	3 (1)
Not confident		4 (1)	0 (0)
Overall *p* value		< 0.0001	
*The room the infant will/does sleep most of the time in relation to parent (n = 333)*			
Planned	n (Col%)	Actual	n (Col%)
Same room	292 (88)	Same room	244 (73)
		Different room	48 (14)
Not sure	9 (3)	Same room	4 (1)
		Different room	5 (2)
Different room	32 (10)	Same room	10 (3)
		Different room	22 (7)
*The place the infant will/does sleep most of the time (n = 349)*			
Planned	n (Col%)	Actual	n (Col%)
Safe place	342 (98)	Safe place	317 (91)
		Unsafe place	25 (7)
Not sure	0 (0)	Safe place	0 (0)
		Unsafe place	0 (0)
Unsafe place	7 (2)	Safe place	6 (2)
		Unsafe place	1 (0)
*The position the infant will/is placed for sleep (n = 347)*			
Planned	n (Col%)	Actual	n (Col%)
Back	326 (94)	Back	294 (85)
		Not Back	32 (9)
Not sure	13 (4)	Back	8 (2)
		Not Back	5 (1)
Not back	8 (2)	Back	6 (2)
		Not Back	2 (1)

^a^Column totals may not equal group N due to missing data

^b^Total column percentage may not equal 100% due to rounding

Despite this confidence, there was a mismatch seen for some infant safe sleep practices between what was prenatally planned and postnatally performed. For example, 88% stated they planned to room share with their baby on their prenatal survey, but only 77% (258/333) reported following this guideline on their postnatal survey, *p* = 0.0067. Similarly, 98% stated prenatally that their baby would be sleeping most of the time in a safe space—a crib, bassinet or play yard. However, the proportion reporting this practice dropped to 93% (323/349) on the postnatal survey, *p* = 0.0013. In addition, 94% reported they planned to place their infant on their back to sleep prenatally but 89% (308/347) stated they were following this safe sleep practice postnatally, *p* = 0.022.

Analysis was done to determine if any demographic factors had higher proportions associated with not following these planned safe sleep practices. See Table 3. Additional pairwise comparisons were then performed for these variables (not shown in Table 3). The proportion of participants who initially stated their child would be sleeping in the same room but had their baby sleep in a different room was higher for non-Hispanic Whites (19%, 45/233) than for other races/ethnicities (5%, 3/59), p = 0.0084, and for participants with incomes of ≥ $75,000 (21%, 36/169) as compared to those with incomes < $75,000 (9%, 10/108), p = 0.0086.

**Table 3:** Comparative analyses of demographics regarding postnatal infant sleep practices of subjects (as per the Postnatal Safe Sleep Survey) who stated on the 28-Week Prenatal Visit Safe Sleep survey that they would be following a practice considered safe (Sleep Location in the same room; Sleep Space in a crib, bassinet, or crib yard; Sleep Position on back)^a,b^

	Sleep location			Sleep space			Sleep position		
	Same/ Same n (Row %)	Same/ Different n (Row %)	*p* Value	Safe/ Safe n (Row %)	Safe/ Unsafe n (Row %)	*p* Value	Back/ Back n (Row %)	Back/ Not Back n (Row %)	*p* Value
Group N	244 (84)	48 (16)		317 (93)	25 (7)		294 (90)	32 (10)	
*Age*			0.82			0.91			0.75
18–24 yrs	29 (88)	4 (12)		36 (92)	3 (8)		33 (94)	2 (6)	
25–29 yrs	75 (81)	18 (19)		100 (92)	9 (8)		91 (88)	12 (12)	
30–34 yrs	81 (84)	15 (16)		105 (93)	8 (7)		102 (91)	10 (9)	
≥ 35 yrs	54 (84)	10 (16)		70 (95)	4 (5)		61 (88)	8 (12)	
*Race/ethnicity*			0.0083			0.31			0.40
NH White	188 (81)	45 (19)		260 (94)	18 (6)		241 (91)	25 (9)	
Hispanic/Latinx	28 (90)	3 (10)		30 (91)	3 (9)		26 (84)	5 (16)	
NH other	28 (100)	0 (0)		27 (87)	4 (13)		27 (93)	2 (7)	
*Family income*			0.18			0.030			0.062
$0–$9,999	9 (90)	1 (10)		11 (100)	0 (0)		10 (91)	1 (9)	
$10,000–$24,999	24 (92)	2 (8)		24 (89)	3 (11)		23 (100)	0 (0)	
$25,000–$49,999	28 (90)	3 (10)		30 (88)	4 (12)		25 (81)	6 (19)	
$50,000–$74,999	37 (90)	4 (10)		43 (86)	7 (14)		41 (87)	6 (3)	
$75,000–$99,999	54 (83)	11 (17)		70 (91)	7 (9)		61 (85)	11 (15)	
≥ $100,000	79 (76)	25 (24)		123 (98)	3 (2)		117 (94)	8 (6)	
Insurance			0.37			0.010			1.00
*Medicare/Medicaid*	54 (90)	6 (10)		58 (88)	8 (12)		54 (90)	6 (10)	
Through employer	181 (82)	39 (18)		249 (94)	15 (6)		230 (90)	25 (10)	
Privately purchased	2 (100)	0 (0)		0 (0)	1 (100)		2 (100)	0 (0)	
*Number of children*			0.097			0.41			0.050
0 Children	91 (79)	24 (21)		129 (93)	10 (7)		115 (88)	16 (12)	
1 Child	85 (83)	17 (17)		111 (95)	6 (5)		106 (96)	5 (5)	
≥ 2 Children	63 (91)	6 (9)		71 (90)	8 (10)		66 (86)	11 (14)	

*NH* non-Hispanic; *yrs* years

^a^Column total may not equal group N due to missing data

^b^Total column percentage may not equal 100% due to rounding

Other data, not shown in Table 3, showed that the proportion of participants initially stating their child would be sleeping in a safe space but who were placing their baby for sleep in an unsafe space was higher for those with incomes < $75,000 (11%, 14/122) as compared to those with incomes ≥ $75,000 (5%, 10/203), *p* = 0.029, and for those with Medicare/Medicaid (12%, 8/66) versus those with employer-provided insurance (6%, 15/264), *p *= 0.01. Subjects with significantly higher percentages of those who initially planned to have their child sleeping on their back but were placing their baby to sleep in other positions included participants with no previous children (12%, 16/131) and those with two or more children (14%, 11/66) as compared to those with one previous child only (4.5%, 5/106), *p* = 0.034 and *p* = 0.018, respectively.

Several other planned and actual infant safe sleep practices were queried of participants (results not shown in a table). High proportions (94+ %) planned and did not have their baby sleep with stuffed animals or pillows. Less than 80% planned on not using a crib bumper at their 28-week visit, but nearly all (97%) reported not using one on their postnatal survey. Although an increased proportion were not using a blanket at the time of their postnatal survey, 10% still reported using one. Only about one-fifth planned on having their baby sleep with a pacifier prenatally but nearly three-fifths reported using one postnatally. About 10% reported they planned to have their baby sleep in bed with them and this proportion was about the same postpartum. Less women (about 75%) were feeding their infant breast milk as compared to those who planned to do so (86%). A small percentage of women reported smoking around their baby (3%) or allowing others to smoke in the home (1%). Like sleep place, space and positioning, there were some mothers who planned to carry out a safe sleep practice but were not doing so postnatally. This includes 6% (20/312) of those who planned to not have their child sleep with a blanket, 5% (16/319) who planned to not have their baby sleep in bed with them and 6% (22/93) who planned to have their infant sleep with a pacifier.

There was evidence of increases in safe sleep knowledge among study participants when comparing pre- versus postnatal survey responses (results not shown in table). For example, of those that stated they were not sure or thought it was unsafe for a baby < 1 year of age to sleep in a play yard on the prenatal survey, almost 90% (28/33) correctly stated it was safe on their postpartum survey. Similarly, of those that stated they were not sure or thought it was safe on their prenatal survey, about two-thirds correctly stated on their postnatal survey that it was unsafe for a baby < 1 year of age to sleep in a car seat in the house (63%, 39/62), a baby swing/bouncy seat (66%, 67/102), or in bed with an adult (65%, 15/23).

Table 4 shows analyses done to determine if there were demographic differences in the proportion of participants who correctly (unsafe) versus incorrectly (safe/not sure) answered the question as to whether it was safe for an infant less than one year to sleep in a car seat in the house, a baby swing/bouncy seat or in bed with an adult on the postnatal survey. Those with incomes < $75,000 had higher percentages stating they were unsure or thought it was safe for a baby to sleep in a car seat in the house than those with greater income. A higher proportion of races/ethnicities other than non-Hispanic Whites, those with incomes < 75,000 and those with Medicare/Medicaid insurance were unsure or stated it was safe for an infant to sleep in a baby swing/bouncy seat. Similarly, there were higher percentages of races/ethnicities other than non-Hispanic Whites and those with lower incomes who were unsure or stated it was safe for an infant to sleep in bed with an adult.

**Table 4:** Comparative analyses by demographics regarding participant’s answer to the question as to whether an infant sleeping in a car seat, a baby swing/bouncy seat in the house, or in a bed with an adult was Correct (unsafe practice) or Incorrect (safe practice/not sure) as reported on the Postnatal Safe Sleep Survey^a,b^

	Car seat			Baby swing/bouncy seat			In bed with an adult		
	Correct n (Row %)	Incorrect n (Row %)	p value	Correct n (Row %)	Incorrect n (Row %)	p value	Correct n (Row %)	Incorrect n (Row %)	p value
Group N	312 (88)	42 (12)		281 (79)	73 (21)		324 (92)	30 (8)	
*Age*			0.9			0.28			0.49
18–24 yrs	37 (90)	4 (10)		30 (73)	11 (27)		37 (90)	4 (10)	
25–29 yrs	100 (88)	14 (12)		85 (75)	29 (25)		104 (91)	10 (9)	
30–34 yrs	101 (87)	15 (13)		96 (83)	20 (17)		110 (95)	6 (5)	
≥ 35 yrs	68 (89)	8 (11)		63 (83)	13 (17)		68 (89)	8 (11)	
*Race/ethnicity*			0.92			0.025			< 0.001
NH White	255 (88)	34 (12)		236 (82)	53 (18)		273 (94)	16 (6)	
Other	57 (88)	8 (12)		45 (69)	20 (31)		51 (78)	14 (22)	
*Family income*			0.0033			< 0.001			0.025
$0–$74,999	103 (82)	23 (18)		83 (66)	43 (34)		110 (87)	16 (13)	
≥ $75,000	195 (93)	15 (7)		183 (89)	27 (13)		198 (94)	12 (6)	
*Insurance*			0.47			0.031			0.074
Medicare/Medicaid	59 (86)	10 (14)		48 (70)	21 (30)		60 (87)	9 (13)	
Other	243 (89)	31 (11)		223 (81)	51 (19)		256 (93)	18 (7)	
*Number of children*			0.42			0.52			0.66
0 Children	125 (86)	21 (14)		113 (77)	33 (23)		132 (90)	14 (10)	
1 Child	110 (91)	11 (9)		100 (83)	21 (17)		113 (93)	8 (7)	
≥ 2 Children	70 (88)	10 (13)		62 (78)	18 (23)		74 (93)	6 (8)	

*NH* non-Hispanic; *yrs* years

^a^Column total may not equal group N due to missing data

^b^Total column percentage may not equal 100% due to rounding

Subjects were asked the degree of agreement or disagreement they had with several statements related to infant sleep on both the pre- and postnatal survey. See Table 5. Significantly higher percentages “strongly agreed” on the postnatal survey as compared to the prenatal survey that it was safe to place a baby to sleep on their back (86% vs 76%, p = 0.0024) and that feeding a baby breast milk reduces the risk of SIDS (44% vs 26%, p < 0.001).

**Table 5:** The degree of agreement or disagreement with statements by participants on the 28-Week Prenatal Visit Safe Sleep Survey as compared to the Postnatal Visit Safe sleep Survey (*N* = 355)^a,b^

		Strongly Disagree	Disagree	Not sure	Agree	Strongly Agree	*p* Value
		n (Row%)	n (Row%)	n (Row%)	n (Row%)	n (Row%)	
1. It is safe to put a baby < 1 year to sleep with stuffed animals	Prenatal	267 (76)	75 (21)	3 (1)	4 (1)	1 (0)	0.1
	Postnatal	2922 (83)	53 (15)	1 (0)	5 (1)	0 (0)	
2. It is safe for a baby < 1 year to sleep in the same bed with adults	Prenatal	214 (62)	101 (29)	8 (2)	19 (6)	3 (1)	0.72
	Postnatal	234 (67)	87 (25)	7 (2)	18 (5)	3 (1)	
3. It is safe for a baby < 1 year to sleep in the same bed with other children	Prenatal	283 (81)	64 (18)	2 (1)	0 (0)	1 (0)	0.42
	Postnatal	299 (85)	49 (14)	2 (1)	0 (0)	1 (0)	
4. It is safe to smoke around a baby	Prenatal	331 (95)	17 (5)	0 (0)	1 (0)	1 (0)	0.74
	Postnatal	327 (93)	22 (6)	1 (0)	0 (0)	1 (0)	
5. It is safe to put a baby down to sleep with loose blankets and/or pillows	Prenatal	295 (84)	52 (15)	0 (0)	3 (1)	0 0)	0.28
	Postnatal	293 (83)	52 (15)	4 (1)	2 (1)	0 (0)	
6. It is safe to put a baby to sleep on their back	Prenatal	1 (0)	3 (1)	8 (2)	71 (20)	265 (76)	0.0024
	Postnatal	1 (0)	2 (1)	1 (0)	45 (13)	302 (86)	
7. It is safe to put a baby to sleep on their side	Prenatal	146 (42)	132 (38)	35 (10)	35 (10)	1 (0)	0.0026
	Postnatal	153 (44)	138 (40)	11 (3)	43 (12)	4 (1)	
8. It is safe to put a baby to sleep on their stomach	Prenatal	216 (62)	97 (28)	20 (6)	12 (4)	2 (1)	0.064
	Postnatal	232 (66)	89 (25)	8 (2)	20 (6)	1 (0)	
9. Feeding a baby breast milk reduces their risk of Sudden Infant Death Syndrome (SIDS)	Prenatal	11 (3)	18 (5)	137 (39)	92 (26)	91 (26)	< 0.001
	Postnatal	8 (2)	15 (4)	70 (20)	102 (29)	156 (44)	
10. Safe sleep practices only apply to nighttime sleep	Prenatal	261 (75)	78 (22)	7 (2)	1 (0)	1 (0)	0.012
	Postnatal	224 (64)	116 (33)	7 (2)	1 (0)	1 (0)	

^a^Row total may not equal group N due to missing data

^b^Total row percentage may not equal 100% due to rounding

Study participants were also asked on their postnatal survey about the sources from which they had heard, seen or read anything about safe sleep. Among the 355 participants, 90% answered healthcare providers, 60% from online sources, 58% from friends/family, 43% from books, 42% from social media, 38% from prenatal classes, 7% from magazines, 5% from TV/Radio and 5% from other sources.

## Discussion

In this study, we found that subject’s confidence in being able to provide their baby a safe place to sleep significantly increased from their 28-week prenatal visit to the 6-week postpartum period. Virtually all (99%) participants stated they were either “confident” or “very confident” on the postnatal survey. Overall, participants demonstrated increases in safe sleep knowledge and in their strength of conviction regarding some safe sleep practices over the study period. Although the proportion of participants using many safe sleep practices at 6-week postpartum was higher than those planning to do so prenatally, there were also a number of safe sleep practices that were being practiced by a smaller percentage of subjects than had planned to practice them at their 28-week prenatal visit.

Similarly, whereas many subjects increased their knowledge and attitudes regarding safe sleep during the study period, some significant deficits remained. For example, demographic groups that had higher proportions that were still unsure or thought it was safe for a baby to sleep in a baby swing/bouncy seat or share a bed with an adult generally included those of race/ethnicity other than non-Hispanic White, with incomes less than $75,000 and/or had Medicaid/Medicare insurance. This observation is consistent with the findings that sleep-related infant deaths have significant racial and ethnic disparities (United States Department of Health and Human Services (US DHHS) 2022). In fact, non-Hispanic Black infants have twice the SUID risk of Non-Hispanic White infants (Parks et al. 2017).

Socioeconomic status is highly correlated with race/ethnicity (Cutter et al. 2003) and lower socioeconomic status has been associated with a higher prevalence of risk factors and deaths due to SUID (Shipstone et al. 2020; Spencer and Logan 2004). Differences in the adherence to safe sleep practices, such as supine positioning, among different racial and ethnic populations may be contributory (Hirai et al. 2019). For example, a meta-analysis found bed sharing to have a nearly three times greater risk of SIDS (Vennemann et al. 2012) and adults sharing beds with infants is more common among African American families (Bombard et al. 2018). Hispanic families also have higher proportions of bed sharing with infants (Parks et al. 2017), and although the national rate of SUID for Hispanic infants is almost half that of non-Hispanic Whites (Parks et al. 2017), a recent study showed that they had SUID rates higher than non-Hispanic White infants in 9 of the 10 largest US cities (Boyer et al. 2022).

Several safe sleep practices (no crib bumper, no blankets, use of a pacifier) had higher proportions of subjects performing them postnatally than were planning to at their 28-week prenatal visit. Still, 10% were using a blanket and over two-thirds were not using a pacifier when placing their infant to sleep 6-week postpartum. Blankets and loose bedding may lead to unintentional suffocation of babies (Colvin et al. 2014; Shapiro-Mendoza et al. 2014; Chowdhury 2017) and increase the risk of SIDS by five times overall and by 21 times if the infant is prone (Scheers et al. 1998; Hauck et al. 2003). Offering a pacifier when placing infants to sleep for both naps and nighttime sleep is recommended (Moon et al. 2022). Meta-analyses and case–control studies have demonstrated the protective effect of pacifiers showing their use decreases the risk of SIDS from 50 to 90% (Hauck et al. 2003, 2005; Mitchell et al. 2006, 1993; Carpenter et al. 2004; McGarvey et al. 2003; Tappin et al. 2002; Arnestad et al. 1997; Fleming et al. 1999; L’Hoir et al. 1999; Li et al. 2006; Vennemann et al. 2009).

A concerning finding was that for several major safe sleep practices, including room sharing without bed sharing, safe sleep space (crib, bassinet, play yard) and supine positioning, there were significantly lower proportions following these practices postnatally than stated they planned to prenatally. Recommendations are that babies sleep in the same room as their parents but on a separate surface designed for infants such as a crib, bassinet or play yard (Moon et al. 2022). Room sharing reduces the risk of SIDS by as much as 50% (Carpenter et al. 2004; Blair et al. 1999; Mitchell et al. 1995, 2017; Tappin et al. 2005). Stomach or side positioning places babies at high risk for SIDS (Hauck et al. 2003; Carpenter et al. 2004; Blair et al. 1999; Li et al. 2003; Fleming et al. 1996; Mitchell et al. 1997), doubling their risk or more (Li et al. 2003). Thus, babies should always be placed on their back to sleep.

It appears lack of knowledge regarding these risk factors did not play a part for these subjects, as they correctly planned to use safe sleep practices. Thus, other factors that we did not explore in the surveys were likely responsible for the lack of safe sleep practice follow through. Demographic groups that had higher proportions who were going to have their child sleep in a safe space but then did not use a crib, bassinet or play yard were those with lower income and insured by Medicaid. This likely indicates that financial limitations may have played a part. In addition, those for whom this infant was their first child and those who had 2 or more previous children had higher percentages that planned to place their child on their back to sleep but weren’t, as compared to those with only one previous child.

Also, of interest, were the demographics of those who stated that they would have their baby sleep in the same room as them prenatally but didn’t. This included demographics not usually considered at increased risk for SUID including non-Hispanic Whites and those with incomes ≥ $75,000. Studies have shown mothers who share a room with their baby wake more frequently and have poorer sleep than those who sleep in another room (Mao et al. 2004; Volkovich et al. 2015). So, it is reasonable to hypothesize that those who have the socioeconomic status and space to have a separate room for their infant to sleep may be more likely to switch their plans regarding this safe sleep practice. However, fewer awakenings and decreased arousal likely contribute to the increased risk of SUID (Phillipson and Sullivan 1978; Kahn et al. 1992; Schechtman et al. 1992; Harper 1986; Kato et al. 2003).

About three-fifths of participants stated they had heard, seen or read about safe sleep both online and from friends/family. Other studies have noted the growing influence of social media and of friends and family members on parental infant sleep practices (Oden et al. 2010; Robida and Moon 2012; Moon et al. 2019). One important factor in changing the number of SUID deaths will be educating not only pregnant mothers but everyone about safe infant sleep practices, in order to improve attitudes and social norms. Few subjects mentioned having heard or read something about safe sleep from magazines or radio/TV, and only just over half read about safe sleep from hand-outs/pamphlets even though all participants had received at least one such item as part of the study.

Although the majority of participants (90%) stated they had heard something about safe sleep from a healthcare provider, this should be 100%. For the medical community to make significant progress in decreasing SUID, it is essential that all healthcare providers provide counsel and always model safe infant sleep guidelines from the start of pregnancy through the completion of infancy. Although everyone needs to be educated, our study identified demographic groups for which targeted and more effective interventions may be needed. However, further studies are needed to determine why some mothers who are knowledgeable about certain safe sleep practices don’t follow through and practice them. Understanding the barriers that contribute to these unsafe decisions will be invaluable in developing effective solutions.

## Limitations

The generalizability of the study may be limited because subjects were from one Midwestern academic center with low diversity in race/ethnicity. In addition, selection bias was likely, as there were significant demographic differences between those who completed both the pre- and postnatal surveys and those who did the prenatal survey only. Like other surveys, our data may be subject to bias because of lack of knowledge, inaccurate recall or social desirability bias. Another limitation is that the survey was not translated into other languages and, thus, we could not include non-English readers. Our survey was limited and did not address all issues related to safe sleep including whether respondents actually had a safe place to put their infant to sleep. With the COVID-19 pandemic developing near the beginning of the study, providers were only offering postpartum visits virtually and our postnatal survey completion rate by text messaging was not as high as we would have expected with in-person visits. Of note, despite limitations, few studies have longitudinally examined pregnant women’s planned practices for infant sleep and compared them to their actual practices postpartum and so our study addresses a significant gap in knowledge.

## Conclusions

Despite increases in safe sleep knowledge and many participant mothers following safer infant sleep practices than had planned to at their 28-week prenatal visit, there were others who had planned on using safe sleep practices but were not doing so after their baby’s birth. Our study identified demographics for which additional targeted safe sleep education and more effective interventions may be needed.

## Data Availability

Data and materials are available to other parties for research purposes after a data sharing agreement plan is agreed to and signed. Those interested should contact the corresponding author.

## Abbreviations

COVID-19: Coronavirus disease 2019
SAS: Previously stood for Statistical Analysis System
SIDS: Sudden infant death syndrome
SUID: Sudden unexpected infant death
TM: Trademark
UIHC: University of Iowa Hospitals and Clinics
US: United States
